# Deciphering Biochemical and Molecular Signatures Associated with Obesity in Context of Metabolic Health

**DOI:** 10.3390/genes12020290

**Published:** 2021-02-19

**Authors:** Daisy Masih, Jitendra Kumar Tripathi, Gurseen Rakhra, Annu Vats, Saroj Kumar Verma, Prabhash Kumar Jha, Manish Sharma, Mohammad Zahid Ashraf, Som Nath Singh

**Affiliations:** 1 Nutrition Division, Defence Institute of Physiology and Allied Sciences, DRDO, Delhi 110054, India; gurseen.bhatia@gmail.com (G.R.); annuvats.nona@gmail.com (A.V.); sirfsaroj@gmail.com (S.K.V.); nutrition-dipas@hotmail.com (S.N.S.); 2 Genomics Division, Defence Institute of Physiology and Allied Sciences, DRDO, Delhi 110054, India; prabhash161@gmail.com (P.K.J.); mohammadzashraf@gmail.com (M.Z.A.); 3 Proteomics Division, Defence Institute of Physiology and Allied Sciences, DRDO, Delhi 110054, India; manishks77@gmail.com

**Keywords:** obesity, prevalence, metabolic syndrome, metabolically healthy obesity, metabolically unhealthy normal weight

## Abstract

This study aims to identify the clinical and genetic markers related to the two uncommon nutritional statuses—metabolically unhealthy normal-weight (MUNW) and metabolically healthy overweight/obese (MHOW) individuals in the physically active individuals. Physically active male volunteers (*n* = 120) were recruited, and plasma samples were analyzed for the clinical parameters. Triglycerides, HDL-Cholesterol, LDL-cholesterol, total cholesterol, C-reactive protein, and insulin resistance were considered as markers of metabolic syndrome. The subjects were classified as ‘healthy’ (0 metabolic abnormalities) or ‘unhealthy’ (≥1 metabolic abnormalities) in their respective BMI group with a cut-off at 24.9 kg/m^2^. Analysis of biochemical variables was done using enzyme linked immunosorbent assay (ELISA) kits with further confirmation using western blot analysis. The microarray was conducted, followed by quantitative real-time PCR to identify and analyze differentially expressed genes (DEGs). The MHOW group constituted 12.6%, while the MUNW group constituted 32.4% of the total study population. Pro-inflammatory markers like interleukin-6, tumor necrosis factor (TNF)-α, and ferritin were increased in metabolically unhealthy groups in comparison to metabolically healthy groups. Gene expression profiling of MUNW and MHOW individuals resulted in differential expression of 7470 and 5864 genes, respectively. The gene ontology (GO) biological pathway analysis showed significant enrichment of the ‘JAK/STAT signaling pathway’ in MUNW and ‘The information-processing pathway at the IFN-β enhancer′ pathway in MHOW. The *G6PC3* gene has genetically emerged as a new distinct gene showing its involvement in insulin resistance. Biochemical, as well as genetic analysis, revealed that MUNW and MHOW are the transition state between healthy and obese individuals with simply having fewer metabolic abnormalities. Moreover, it is possible that the state of obesity is a biological adaptation to cope up with the unhealthy parameters.

## 1. Introduction

Adipocytes (classically known for the storage of excess fat and lipids) have now emerged as an active endocrine organ [1]. In addition to it, there is an increased level of hormones like leptin, resistin, and cytokines in obese subjects compared to non-obese individuals, which cumulatively relates obesity to various co-morbidities [2,3,4]. Surprisingly, not all obese people show increased adipokines levels, and instead, show a healthy profile like a normal-weight individual. The state of obesity showing an absence of the metabolic complication and BMI greater than 30 kg/m^2^ has been named as metabolically healthy obesity (MHO) [5]. On the other hand, the metabolic abnormality can be observed in normal-weight individuals who showed a risk of development of metabolic syndrome with their BMI lying within a healthy normal range. These individuals are called as metabolically unhealthy normal-weight (MUNW) [6].

Though numerous studies have been published related to metabolic health, and many are undergoing; still, no single defined criteria to predict metabolic health is available [7]. Alternatively, Karelis put forward an idea of the initial set of simple biochemical estimations as markers to identify the MHO individual based on their research studies and suggested if four out of five metabolic markers are in the normal range, a person can be considered as MHO [8]. The five metabolic markers in their study were triglycerides (TG), HDL-Cholesterol (HDL-C), LDL-cholesterol (LDL-C), total cholesterol (TC), and insulin resistance.

Dietary pattern, physical inactivity, and a sedentary lifestyle contribute to obesity, though; one should also embark on the influence of genes in the body [9]. The genetic predisposition to obesity has a vital role in the development of obesity subtypes. Some genes, like *FTO* and *SH2B1*, are genetically thrifty genes, known to be naturally selected for obesity [10,11,12]. On the other hand, the genetic contribution of pathways involved in adipose tissues also informs us about the development of MHO and MUO phenotypes.

Therefore, taking into account simple biochemical markers, we planned our study with the criteria of MHO proposed by Karelis, 2004, with some modifications [8]. In the present study, we aimed to analyze the prevalence of metabolically healthy overweight/obesity (MHOW) and metabolically unhealthy normal-weight (MUNW) individuals among young, physically active Indian males belonging to the same region, ethnicity, age, and dietary habits. Moreover, understanding the metabolic profile of such individuals is obscure and requires to be explored with more research; therefore, whole-body composition analyses with hematological parameters and biochemical estimations of hormones and inflammatory markers along with whole-genome expression profiling have been studied in this research article. Since genetic insight into MHOW and MUNW is not studied on physically active individuals yet, this makes our study as distinct and novel.

## 2. Materials and Methods

### 2.1. Subjects

The study was conducted on young male volunteers (age ≥ 18) of a fitness center in Karnal, Haryana. Participants were indulged in exercise for a minimum tenure of 6 months and were moderately active. They were explained with the study protocol approved by the Institutional Ethics Committee (IEC DIPAS, IEC/DIPAS/C-1/2 DATED 26.5.15). A total of 120 individuals consented in written for participation in the study. Dietary intake was assessed using a 7-day self-filled dietary intake form. These contained different food items as study participants were free living. Although this method suffers limitations as both over- and under-reporting of food intake are known problems [13,14], calorie and nutrient intake was computed from the dietary recalls based on values reported for Indian recipes using the database of National Institute of Nutrition (http://218.248.6.43:8080/CountWhatYouEat/, accessed on 1 July 2020) and dietary manual (https://www.nin.res.in/downloads/DietaryGuidelinesforNINwebsite.pdf, accessed on 1 July 2020) [15]). Out of 120 participants, 24 male participants (six participants per group) with an average age of 23.8 years were picked up for further microarray analysis. Healthy volunteers with a minimum 18 years of age, engaged in more than six months of regular exercise constituted inclusion criteria, while individuals with long term medication, due to acute or chronic illness, were excluded from the study.

The Criterion for Metabolic syndrome (Karelis, 2004, with some modifications) [8]:

- Fasting TG level ≥ 150 mg/dL
- Fasting HDL-C ≤ 40 mg/dL (men)
- Fasting LDL-C ≥ 100 mg/dL
- Fasting TC ≥ 200 mg/dL
- C-reactive protein ≥ 1.18 (90th percentile)
- Insulin resistance (McAuley’s Index) ≥ 15.76 (90th percentile)

Thus, taking BMI ≥ 24.9 kg/m^2^ as overweight/obese and the above-mentioned metabolic syndrome, we divided the population into four groups considering zero metabolic syndromes for metabolically health profiles.

Categorization of participants:

- Metabolically healthy normal-weight (MHNW): BMI ≤ 24.9 kg/m^2^, with no markers of metabolic syndrome
- Metabolically unhealthy normal-weight (MUNW): BMI ≤ 24.9 kg/m^2^, with one or more markers of metabolic syndrome
- Metabolically healthy overweight/obese (MHOW): BMI ≥ 25 kg/m^2^, with no markers of metabolic syndrome
- Metabolically unhealthy overweight/obese (MUOW): BMI ≥ 25 kg/m^2^, with one or more markers of metabolic syndrome

### 2.2. Anthropometric Measurements

Height was measured using a measuring rod with the least count as 0.1 cm (Seca 216, Seca Asia Pacific medical measuring systems and scales, Kuala Lumpur, Malaysia). Bodyweight was measured with bare feet, in light clothing using a bioelectric impedance analyzer (Tanita BC-420MA, body composition analyzer, Tanita Corporation, Tokyo, Japan). Body mass index (BMI) was calculated using the formula weight in kilograms divided by squared height in meters, i.e., Weight (kg)/Height (m^2^). Body composition analysis was done before breakfast between 0700 h and 1000 h in the post-absorptive state.

### 2.3. TEE, PAL Value, and BMR Calculation

Total energy expenditure (TEE) for physical activity was monitored using accelerometry-based wearable Actical^®^ devices (Respironics, mini mitter co. Inc., Bend, OR, USA). The volunteers wore the device on the wrist for 7 days continuously to record minute by minute energy expenditure [16]. BMR was calculated manually using the prediction equation for Indians [17]) (Supplementary Table S1). Further, physical activity level (PAL) was calculated by the division of total energy expenditure (TEE) by the basal metabolic rate (BMR) (PAL = TEE/BMR).

### 2.4. Collection of Blood Samples

Whole venous blood was collected in plain EDTA and heparinized vacutainers by venipuncture in the arm. Hematological parameters were measured on a fully automatic hematology analyzer (MS4e, MeletSchloesing Lab., Osny, France). Plasma was collected after centrifugation of heparinized whole blood sample for 10 min at 3000 rpm, while serum was isolated after keeping the whole blood sample at room temperature for about 1 h. Serum samples were utilized for evaluating hormones and biochemical variables, while human plasma was conserved with a protease inhibitor for immunoblot analysis. Both the samples were stored at −80 °C. For microarray of whole-genome genes, 2.5 mL of whole blood was drawn by venous puncture directly into PAXgen blood RNA tubes (BD, Franklin Lakes, NJ, USA) to stabilize RNA and stored at −80 °C for further use.

### 2.5. Analysis of Biochemical Variables

Commercially available kits were used for the analysis of lipid profile, i.e., total cholesterol (Randox lab ltd., County Antrim, UK), triglycerides, high-density lipoprotein-cholesterol (HDL-C), and low-density lipoprotein- cholesterol (LDL-C) (Agappe diagnostics ltd, Kolkata, India). ELISA kits were used for insulin (Sigma Diagnostics Inc., Livonia, MI, USA), leptin (Diagnostics Biochem Canada Inc., London, ON, Canada), adiponectin (Elabscience Biotechnology Co. Ltd., Wuhan, China), C-reactive protein (CRP, Sigma Diagnostics Inc., Livonia, MI, USA), interleukin-6 (IL-6, Diaclone SAS, Besançon, France), tumor necrosis factor-α (TNF-α, Diaclone SAS, Besançon, France), ferritin (Bio-detect, Laguna Hills, CA, USA) as per assay manual of the kits. Insulin resistance was calculated using McAuley’s Index i.e., exp (2.63–0.28 ln insulin [μU/mL]–0.31 ln triglycerides [mM/mL]) [18].

### 2.6. Antibodies

The primary antibodies used were primary rabbit polyclonal anti-Leptin (1∶1000, Ob (A-20) sc-842), primary rabbit polyclonal anti-AdipoR2 (1∶1000, (H-44) sc-99184), primary rabbit polyclonal anti-IL-6(1∶1000, (H-183) sc-7920), primary rabbit polyclonal anti-β-actin (1:1000 (N-21) sc-130656) from Santa Cruz Biotechnology, Inc (Heidelberg, Germany). Primary monoclonal mouse anti-ferritin (1∶1000 (E63C02001)), primary rabbit anti-TNF-α (1:500, E18-7014-1) from EnoGene Biotech Co., Ltd., New York, NY, USA. The secondary antibody used was goat anti-rabbit IgG-HRP (sc-2004, 1:25,000) and goat anti-mouse IgG-HRP (sc-2004, 1:25,000) of SantaCruz Biotechnology, Santa Cruz, CA, USA.

### 2.7. Immunoblot Analysis

Sample containing 30 µg protein was loaded on 10% SDS-PAGE and transferred to PVDF membrane on the semi-dry transfer unit (Bio-Rad laboratories, Hercules, CA, USA). The blots were incubated with primary antibody for the protein of interest for 3 h followed by the addition of IgG-horse radish peroxidase conjugated secondary antibody against the specific primary antibody for 2 h at room temperature. Immunoreactive bands were visualized with a gel documentation instrument (Alliance Q9 Advanced, UVITECH Chemiluminescence Documentation Systems), and densitometric analysis was done using Image J analysis software.

### 2.8. RNA Extraction

The PAXgen blood RNA tubes containing samples were thawed for two hours at room temperature to ensure the whole cells lysis. Total RNA was isolated using PAXgene Blood RNA Kit (Qiagen, Valencia, CA, USA) following the manufacturer’s guidelines, and extracted RNA was stored at −80 °C.

### 2.9. Oligonucleotide Microarray Hybridization

The quality and concentration of RNA were checked using a spectrophotometer (NanoDrop2000, Thermo Scientific, Wilmington, DE, USA), and RNA integrity was assessed using Agilent 2100 Bioanalyzer system (Agilent Technologies Inc., Lexington, MA, USA). The RIN values more than 7 were considered adequate for hybridization and scanning. A total of 250 ng of high-quality RNA was used to synthesize complementary DNA followed by an invitro transcription step in which amplification and labeling were done to produce biotin-labeled cRNA according to the MessageAmp II a RNA Amplification kit (Ambion, Inc., Austin, TX, USA) as recommended by Illumina′s sample labeling procedure. The microarray and hybridization were performed on Illumina HumanHT-12 v4 Expression BeadChip (Illumina, Inc., San Diego, CA, USA). The total fluorescence emission from a single spot is collected as a total signal intensity, which is directly proportional to the degree of hybridization [19].

### 2.10. Gene Expression Profiling and Gene Ontology(GO)

Differentially expressed genes (DEGs) were identified using Genome Studio™ Gene Expression Module v 1.0 (Illumina Inc., San Diego, CA, USA) and statistically enriched with fold change ≥ 2.0 and *p*-value ≤ 0.05. Functional gene ontology and pathway analysis were performed using online software Enrichr [20]. *p*-value ≤ 0.05 was adjusted using the hypergeometric distribution. Enriched pathways network was constructed using Cytoscape 3.2.1 [21] with different color coding and shapes for up- and downregulated genes and processes for symbolic representation of the molecular links between genes and processes. The network-based analysis was performed using Network Analyst [22], which provides visual and statistical analytes for gene expression analysis to obtain highly interconnected hub nodes [23].

### 2.11. Functional Gene Set Enrichment Analysis (GSEA) and Upstream Regulation of Shared DEGs

To distinguish the inference of shared DEGs, we executed a functional analysis using the Enrich R platform [24]. The annotation of the significant gene list was extensively assessed with this software using various libraries, such as Gene ontology library64, biocarta, Kyoto Encyclopedia of Genes and Genomes pathway (KEGG), Wikipathway, Reactome pathway, and Panther. The analysis was based on *p* < 0.05 and performed with a Fischer Exact test. Upstream and kinase enrichment analysis were used to prioritize transcription factors and protein kinases by using Expression2Kinase (X2K) bioinformatic tool.

### 2.12. Validation of Some Candidate Genes Responsible for Obesity by Real-Time-PCR

Among the study population, five samples for each group of MUNW, MHOW, and MUOW with matched age, ethnicity, and occupational routine were selected for validation of the obtained microarray data by reverse transcription-polymerase chain reaction (RT-PCR) for five DEGs. *CUL1* (Cullin 1) was selected from hub gene analysis, *G6PC3* (Glucose-6-phosphatase catalytic subunit 3) and *RHEB* (Ras Homolog Enriched In Brain) were selected from the insulin resistance pathway, while *STAT3* (Signal Transducer And Activator Of Transcription 3) and Amyloid β A4 precursor protein-binding family B member 1-interacting protein (*APBB1IP*) were selected from the leptin pathway. Further confirmation of DEGs acquired from microarray was executed using two-step RT-PCR on Biorad CFX96 (Hercules, CA, USA). β-actin was used for normalization of selected candidate DEGs.

### 2.13. cDNA Synthesis and Quantitative Real-Time Quantitative PCR (qRT-PCR)

cDNA synthesis was performed using a commercially available kit (PrimeScript ™ 1st cDNA Synthesis Kit, Clontech Laboratories, Inc., A Takara Bio company) following the manual provided by the manufacturer. The synthesized cDNA was diluted 10 times for utilization for real-time PCR. Briefly, the 1 µL cDNA was mixed with 0.5 µL TaqMan probe and 5 µL mastermix. The volume of the reaction mixture was made up to 10 µL with Rnase free water. To validate the microarray findings, the expression levels of genes were quantified relative to the endogenous control gene, β-actin (ACTB), using pre-designed TaqMan gene expression assays (Applied Biosystems, Foster City, CA, USA).The mean fold change for each sample was calculated by using the 2DDCt method. The cDNAs were confirmed using 2.5% agarose in agarose gel electrophoresis.

### 2.14. Statistical Analysis

Statistical analyses were performed using Graph Pad Prism version 5.0 software for Windows (Graph pad Prism software, Laolla, CA, USA) with the level of statistical significance set at *p* ≤ 0.05. Parameters were expressed as mean ± standard deviation. One wayanalysis of variance (ANOVA) followed by post hoc Bonferroni test was made for comparison between the four groups.

## 3. Results

### 3.1. Prevalence of Metabolically Unhealthy and Healthy Phenotypes

**Figure 1:**
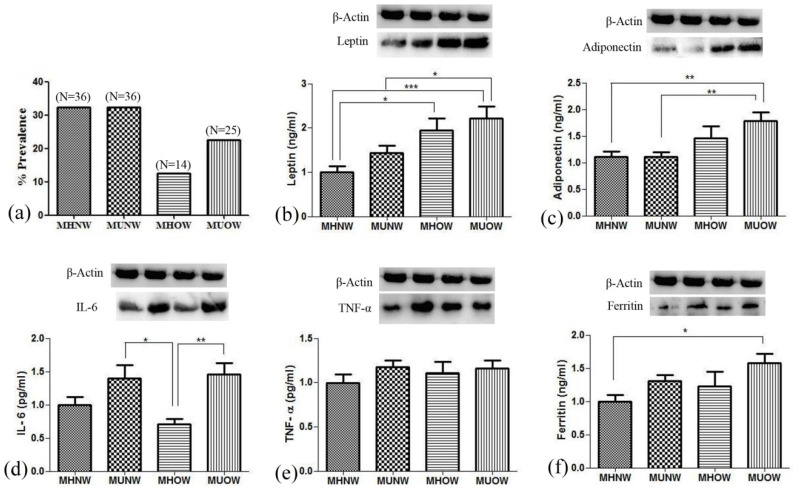
Figure showing percent prevalence of nutritional status in study population (**a**), and levels of hormones and inflammatory markers present in the blood among different groups analyzed by ELISA and Western blotting (**b**) leptin; (**c**) adiponectin; (**d**) IL-6; (**e**) TNF-α; (**f**) ferritin; values are expressed as mean ± SD with * *p* < 0.05, ** *p* < 0.01, *** *p* < 0.001.

The mean age, height, BMI, and other anthropometric parameters are described in Table 1. Out of 120 individuals recruited for the study, only 111 were considered for the analysis. The other 9 participants could not be included due to less blood samples and incomplete data. The varying nutritional status showed a different prevalence of obesity in the study population. Out of 111 participants, 39 were overweight and obese, consisting of 35.1% of the sample, while the rest of the population (64.9%) consisted of healthy individuals. Taking into account metabolic health of the participants, 35.8% (*n* = 14) and 64.2% (*n* = 25) individuals were categorized as metabolically healthy overweight (MHOW) and metabolically unhealthy overweight (MUOW), respectively. In comparison, 50% (*n* = 36) of the normal-weight individuals were categorized as metabolically unhealthy normal-weight (MUNW). The prevalence of all the nutritional statuses is presented in Figure 1a.

### 3.2. Analysis of Clinical and Biochemical Parameters

**Figure 2:**
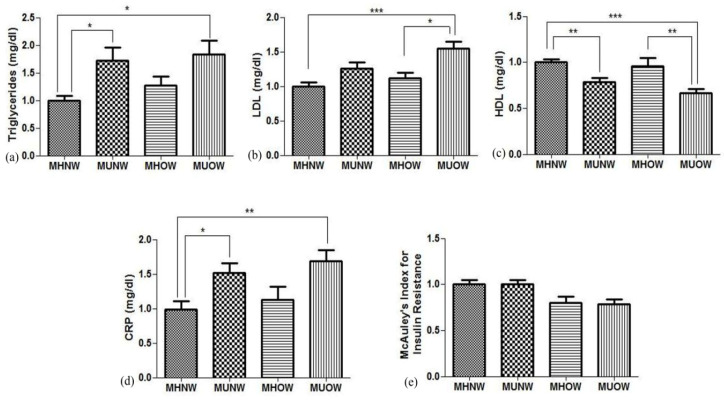
Figure showing ELISA estimation for metabolic syndrome markers (**a**) triglycerides, (**b**) low density lipoprotein (LDL), (**c**) high density lipoprotein (HDL), (**d**) C-reactive protein (CRP), and (**e**) McAuley′s Index for insulin resistance and values are expressed as mean±SD with * *p* < 0.01, ** *p* < 0.01, and *** *p* < 0.001.

Hematological profile analysis depicted an elevation in the blood levels of white blood cells (WBC), thrombocytes, and red blood cells (RBC) of the metabolically unhealthy group in contrast to metabolically healthy groups (Table 2).MHOW participants had proximate fat mass and fat free mass to MUOW, while the former group had significantly lower levels of TG (77.2 ± 38 mg/dLvs.112 ± 74 mg/dL) and LDL (68.8 ± 16 mg/dL vs. 95.3 ± 31 mg/dL) with a significantly higher HDL (57.1 ± 19.6 mg/dL vs. 39.5 ±14 mg/dL). The MUNW group showed similar metabolic characteristics as the MUOW group with high levels of TG (108.1 ± 82 mg/dL) and LDL (77.4 ± 32 mg/dL) and low levels of HDL (46.4 ± 16 mg/dL) (Figure 2). Figure 2 represents clinical data related to the metabolic risk factors, including TG, HDL-C, LDL-C, CRP, and insulin resistance. Since inflammation is coupled with obesity, inflammatory markers were studied. Leptin and adiponectin, in addition to interleukin (IL)-6, tumor necrosis factor (TNF)-α, and ferritin, were analyzed by ELISA, as well as immunoblotting, followed by densitometry (Figure 1b–f). The levels of leptin and adiponectin were elevated in MHOW and MUOW in comparison to normal-weight groups, while IL-6 was low in MHOW compared to MUNW and MUOW.

### 3.3. Microarray Analysis

#### 3.3.1. Identification of DEGs among Different Metabolic Health Subtypes w.r.t. Metabolically Healthy Normal-Weight

In order to identify DEGs in the MUNW, MHOW, and MUOW groups, the processed data were loaded into Genome Studio™ Gene Expression Module, taking MHNW as a reference. The total DEGs in MUNW, MHOW, and MUOW were 12,885, 13,355, and 13,671, respectively. On screening the DEGs based on two-fold change at *p*-value ≤ 0.05, whole-genome microarray analysis resulted in 7470, 5864, and 12 DEGs for the MUNW, MHOW, and MUOW groups, respectively. Since only 12 genes were obtained for MUOW after applying fold change and significance level, further bioinformatics analysis was carried out only for MUNW and MHOW w.r.t MHNW. Table 3 and Table 4 represent the top 5 upregulated and downregulated DEGs in the MUNW and MHOW groups, respectively, in comparison to the MHNW group.

#### 3.3.2. Functional Gene Set Enrichment Analysis (GSEA) and Upstream Regulation of Shared DEGs

To analyze the significantly overexpressed biological pathways and gene ontology terms in the DEGs acquired from the groups, GSEA was performed. In MUNW, Panther pathway enrichment suggested ‘JAK-STAT signaling’ pathway (P00038, *p*-value: 0.03), and Reactome pathway enrichment resulted in the ‘translation’ pathway (R-HSA-72766, *p*-value: 0.002) as overexpressed GO biological pathway. GO term enrichment suggested cotranslational protein targeting to the membrane (GO:0006613) with adjusted *p*-value 0.007 as the most overexpressed GO term. Table 5 and Table 6 represent the top significantly enriched biological pathways and GO terms in group MUNW. In MHOW, Biocarta pathway enrichment suggested ‘The information-processing pathway at the IFN-β enhancer’ pathway (h_pcafpathway, *p*-value: 0.05) and KEGG pathway enrichment showed ‘Ribosome’ pathway (hsa03010, *p*-value: 0.016) as overexpressed GO biological pathway. GO term enrichment suggested ‘gene expression’ (GO:0010467) with adjusted *p*-value 0.001 as the most overexpressed GO term. Table 7 and Table 8 represent the top significantly enriched biological pathways and GO terms in group MHOW.

The upstream analysis resulted ‘Aryl hydrocarbon receptor (AHR)’ and ‘Leucine-rich repeat kinase 2 (LRRK2)’ as highly ranked transcription factor and regulatory kinases, respectively, in MUNW, while a total of seven transcription factors were significantly expressed—in which estrogen receptor 1(ESR1) was highly ranked, while lymphocyte cell-specific protein-tyrosine kinase (LCK) was highly ranked protein kinase in MHOW. Supplementary Tables S2 and S3 represent the transcription factors and Kinases for MUNW, respectively, and Supplementary Tables S4 and S5 represent the transcription factors and Kinases for the MHOW groups, respectively, in comparison to the MHNW group.

### 3.4. Identification of Hub Genes by Network-Based Analysis

**Figure 3:**
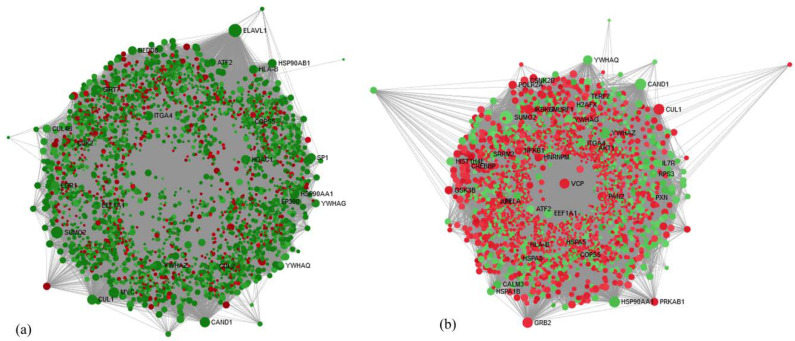
Network-based hub gene analysis of the (**a**) MUNW and (**b**) MHOW groups in comparison to control group, MHNW using Network Analyst online tool Dot designation: Green—Downregulated, Red—Upregulated. The size of node depicts the interaction of the genes with other genes in terms of the degree. The larger the red dot, the more important and regulated pathways in comparison to MHNW.

The network-based analysis was performed using the online tool, NetworkAnalyst, to identify important and most interconnected genes called as hub genes among the DEGs obtained from the data of two groups. In the MUNW group, ELAV Like RNA Binding Protein 1 (*ELAV1*), was a highly ranked hub gene with 753 degrees and 1634,759.12 betweenness centrality showing −3.37-fold change. In the MHOW group, Small Ubiquitin-Like Modifier 2 (*SUMO2*) was a highly ranked hub gene with 425 degrees and 616,100.7 betweenness centrality showing −2.33-fold change. Network-based hub genes analysis is presented for the MUNW and MHOW groups in Figure 3a,b. Supplementary Tables S6 and S7 represent the top 10 hub genes for the MUNW and MHOW groups.

### 3.5. Identification of Network Pathways

**Figure 4:**
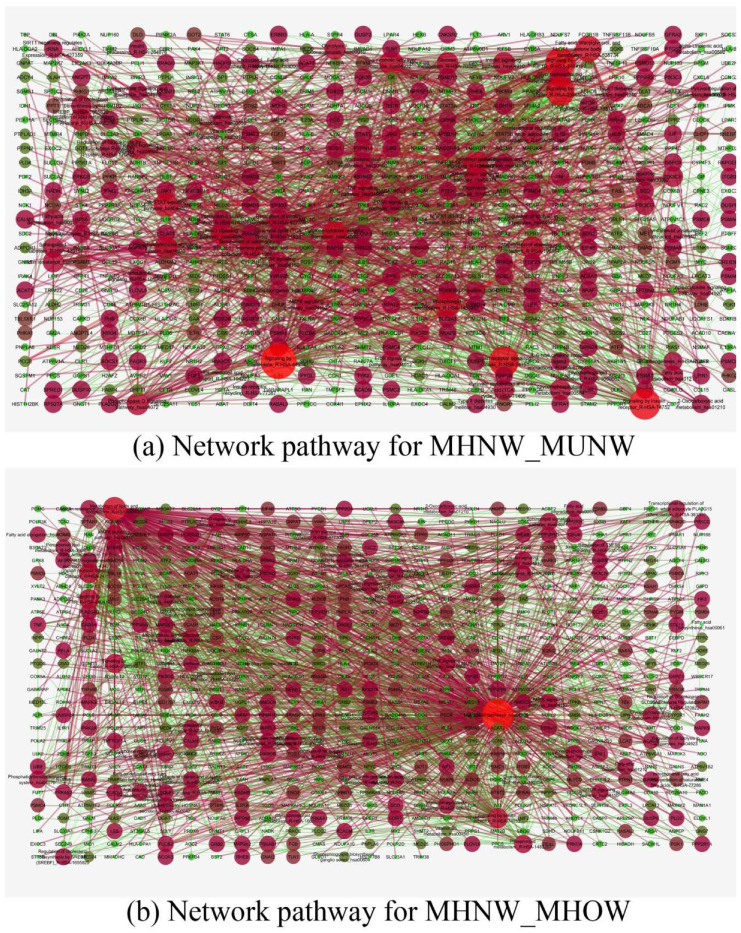
Nutritionally important Network pathways of the (**a**) MUNW and (**b**) MHOW groups in comparison to control group, MHNW. Dot designation: Green –Downregulated, Red-Upregulated. Thesize of node depicts the interaction of the genes with other genes in terms of the degree. The larger the red dot, the more important and regulated pathways in comparison to MHNW.

Nutritionally important pathways were selected, and DEGs of those pathways were input into the Cytoscape software for network analysis. In MUNW, signaling by interleukins showed the highest degree with 109 genes (closeness centrality—0.31, betweenness centrality—0.134) with Phosphoinositide-3-Kinase Regulatory Subunit 2 (*PIK3R2*) showing the highest degree [20] among genes, while in MHOW, metabolic pathways showed the highest degree with 314 genes (closeness centrality—0.442, betweenness centrality—0.606) with Phosphatidylinositol-4,5-Bisphosphate 3-Kinase Catalytic Subunit β (*PIK3CB*) showing the highest degree [16] among genes. Figure 4a,b represents the network of pathways made by using nutritionally important genes for MUNW and MHOW, respectively. Also, the nutritionally important genes of the respective groups are listed in Supplementary Tables S8 and S9.

### 3.6. Validation of Genes by Real-Time PCR

**Figure 5:**
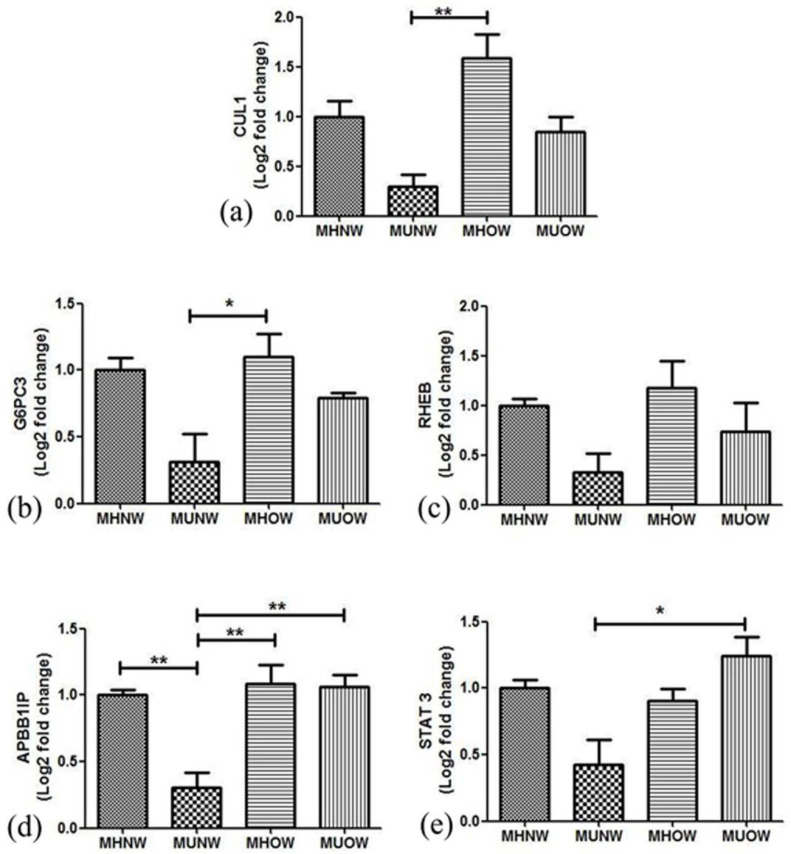
Fold changes in mRNA expression tested by qRT-PCR in different metabolic health groups in comparison to MHNW (**a**) *Culin 1*, (**b**) *G6PC3*, (**c**) *RHEB*, (**d**) *APBB1IP*, (**e**) *STAT3*.* *p* < 0.05, ** *p* < 0.01.

To validate the results of the microarray analysis, some genes were quantitatively analyzed by real-time PCR after technical verification. Though bioinformatics analysis was done only with the MUNW and MHOW groups, we still tried to analyze the expression of the selected genes in the MUOW group for confirmation. For biological validation, 20 samples were analyzed independently for microarray results. These samples were other than those used for microarray analysis, but from the same group (5 samples each for MUNW, MHOW, MUOW). The expression levels of five candidate genes (*CUL1, APBB1IP, STAT3, RHEB,* and *G6PC3*) were validated using qRT-PCR (Figure 5). The mRNA expression of the selected genes as tested by qRT-PCR showed similar results as microarray though the results did not estimate true fold changes.

A significant difference in the expression pattern of *CUL1*, *APBB1IP*, and *G6PC3* was observed between MUNW and MHOW. However, the altered expression was not significant between the MHNW and MUNW or MHOW group. β-actin was used as the endogenous control for normalization of candidate gene expression. Figure 5 depicts the fold change of mRNA expression of selected candidate genes in the MUNW, MHOW, and MUOW groups using quantitative real-time PCR in comparison to MHNW.

## 4. Discussion

Although obesity is a disease, not every obese individual is unhealthy or shows metabolic risk markers. Some obese individuals are shielded against the cardiometabolic risk factors, and hence, called metabolically healthy overweight/obese (MHOW/MHO). Similarly, some healthy individuals show metabolic risk markers even though their BMI was within the range of healthy person, and hence, called metabolically unhealthy normal-weight (MUNW) individuals. The mechanisms underlying the advancement of MHOW/MHO and MUNW are still poorly understood. Even though many studies are conducted using omental or subcutaneous adipose tissue to state a variety of pathways involved and affected in MHO and MUNW [25,26,27], the lack of genetic insight using whole blood to study the genetic expression in various pathways majorly in two uncommon obesity, i.e., MUNW and MHOW needs to be explored which makes this study novel itself.

Due to the unavailability of a universally accepted definition of metabolically healthy obesity (MHO), many studies considered 0, 1, or 2 metabolic syndrome components with or without insulin resistance for defining MHO criterion [6,28,29,30,31,32,33]. Therefore, it had been suggested that MHO is a transition state between MHNW and metabolically unhealthy obesity (MUO) with simply having fewer metabolic abnormalities. Some studies have also suggested that obesity is a biological adaptation leading to changes in adipose tissue biology in response to weight gain [34,35]. Considering the zero metabolic syndrome criterion, the present study explores metabolically healthy and unhealthy phenotype. The prevalence of MHOW individuals in our study is comparable to an Indian study published by Geetha etal.2011 [28]. The haphazard prevalence of metabolically healthy and unhealthy profiles ranges widely, with 10% to 47.7% all over the world [33,36,37,38,39,40]. Such arbitrary results of prevalence may be due to varied country, region, age, gender, race, screening techniques, and absence of a distinct criterion for metabolically healthy and unhealthy individuals [41].

The development of a pathophysiological state in a body modulates the overall transcriptome, which results in varying genetic expression. Microarray helps in global transcriptome analysis to explore the biological insights into various diseases pathogenesis. We used an end-to-end approach starting from the evaluation of whole blood gene expression profile in MUNW, MHOW, and MUOW with matched controls (MHNW), followed by integrated bioinformatics analysis to extract novel biological genes and pathways and finally, autonomously validated significantly altered common DEGs by real-time PCR. To our surprise, on applying filters of 2-fold change and *p*-value ≤0.05, MUOW left with only 12 genes out of 13,671 DEGs; therefore, we continued the further functional analysis with two groups (MUNW and MHOW). Since the volunteers were into regular physical activity, their metabolic state may have switched from MUOW to MHOW, due to which the expression of genes were not in required fold change. On reviewing the literature, we found that a person’s nutritional status can switch between metabolically healthy and metabolically unhealthy status and vice versa [42,43]. A 10-year follow-up study of North West Adelaide also reported the transition of metabolically unhealthy obese MUO to MHO [44]. Thus, it is stated that metabolic health is not a steady-state and can be preserved by targeted interventions [42].

Interestingly, we got 7470 and 5864 DEGs in MUNW and MHOW, respectively, under the significance threshold of adjusted *p*-value ≤ 0.05 and fold change ≥ 2 compared to the healthy control. In MUNW, the gene enrichment analysis showed overexpression of GO-biological pathway ‘JAK-STAT signaling’ in the Panther database. The JAK-STAT signaling pathway is activated by cytokines and interleukins, which further forms STATs dimers and changes the gene expression in the nucleus upon translocation [45,46]. The plasma levels of IL-6, TNF-α, and ferritin in the study participants were high in metabolically unhealthy profile as also reported by many studies [47], suggesting the implication of the JAK-STAT signaling pathway. On the other hand, Biocarta enrichment analysis showed overexpression of ‘The information-processing pathway at the IFN-β enhancer’ pathway, which suggests chromatin remodeling resulting in the activation of transcription, due to interferon-β [48,49]. The other enriched pathways analysis revealed common pathways in both the groups (MUNW and MHOW), such as ‘Translation’, ‘Ribosome’, ‘SRP-dependent cotranslational protein targeting to membrane’, ‘Major pathway of rRNA processing in the nucleolus’, ‘rRNA processing’. These outcomes imply that the two groups are varying from each other in some parameters; however, they showed similarity in their gene expression at the translation level as also depicted by GO term enrichment. This suggests that MUNW and MHOW are only biological adaptations to cope up with unhealthy parameters, and they are intermediate states between MHNW and MUO nutritional status.

Next, we analyzed the overexpressed transcription factors via upstream analysis, which showed upregulation of the ‘Aryl hydrocarbon receptor (AHR)’ transcription factor in the MUNW group. AHR plays a crucial role in obesity metabolism by promoting adipogenesis, and it is reported that AHR inhibition leads to reversal of obesity and hepatic steatosis in mice [50]. In contrast, the upstream analysis showed overexpression of ‘Estrogen receptor 1(ESR1)’ transcription factor in the MHOW group. ESR1 gene polymorphism exhibited a reduced BMI in male and female subjects and also known to attenuate the risk of obesity [51,52]. ESR1 forms complex with 17-β estradiol (E2) (E2/ESR1) and modulates adipose tissue vascular endothelial growth factor A (VEGFA), thereby helping in angiogenesis, attenuation of inflammation, and ameliorating adipose tissue function [53]. Thus, the upregulation of AHR and ESR1 gives us a lead signal to variant metabolic profile in MUNW and MHOW, respectively.

To gain additional genetic insight towards the nutritionally linked significant pathway in MUNW and MHOW, the nutritionally important pathways were selected and analyzed. Two nutritional network pathways that were significantly enriched in MUNW and MHOW subjects were ‘signaling of interleukins’ and ‘metabolic pathways’. Interleukins are produced by leukocytes and target the sites in a paracine and autocrine manner. The oligomerization of interleukins activates various cascades, including the JAK-STAT signaling pathway and MAPK pathway [54]. The ‘metabolic pathways’ involve enzyme-mediated chemical reactions that lead to catabolism and anabolism in the body. Thus, ‘signaling of interleukins’ portrays the major involvement of inflammatory and anti-inflammatory processes regulating in MUNW as also shown by plasma levels of inflammatory markers in the study participants, while the highly regulated metabolic pathways in MHOW portrays the normal involvement of various regulatory processes. The nutritionally important genes ‘*PIK3R2*’ and ‘*PIK3CB*’ in the MUNW and MHOW groups, respectively, are a regulatory and catalytic components of Phosphoinositide-3-Kinase (PI3K), a lipase kinase. The regulatory role of *PIK3R2* was reported as ameliorating insulin sensitivity in *PIK3R2* knockout mice [55], while a promoter variant of *PIK3CB* is reported to provide protection from insulin resistance in obese and non-obese individuals [56]. These results imply the important role of *PIK3R2* and *PIK3CB* in regulating insulin sensitivity in obese and non-obese individuals.

The disease pathophysiology can be best understood by analyzing the level of gene expression of the DEGs overlapped between the groups; therefore, we selected genes from hub gene analysis, insulin, and leptin pathways. Among Hub genes, Cullin1 (*CUL1*, fold change expression of MHNW = 1.00, MUNW = 0.29, MHOW = 1.56, and MUOW = 0.85) is crucial for cell scaffolding and ubiquitin associated proteolysis [57]. Moreover, *CUL1* (along with SKP1 (S-phase-kinase-associated protein 1) and F-Box protein) forms the largest E3 ubiquitin ligase family (Skp1-Cullin1-F-box (SCF)) E3 ligase is known for controlling obesity with an association of Skp2 [58]. It is reported that the mice knockout for SCF Fbxo40 shown elevated levels of IRS1 of the insulin signaling pathway and play a vital role in insulin resistance [38]. Thus, the overexpression of *CUL1* in MHOW and under-expression in MUNW suggests the diverse nature of MHOW individuals. Among other DEGs, overexpression of the *APBB1IP* gene was quantified (fold change expression of MHNW = 1.0, MUNW = 0.32, MHOW = 1.18, and MUOW = 0.74). *APBB1IP* is commonly known as Rap1-GTPase, and plays a vital function in diet-induced obesity, insulin, and leptin resistance. Genetic ablation of Rap1-GTPase shields against dietary obesity and imbalance of glucose, insulin, and leptin sensitivity, reduction in inflammation, and ER stress in the hypothalamus [59]. The overexpression of the *APBB1IP* gene in MHOW in comparison to MUNW suggests leptin resistance as also shown by ELISA and Western blotting of leptin. Moreover, it is reported decreased values of McAuley’s index corresponded to increased insulin resistance, which is also shown by this study [60].

Taking into account insulin pathway and insulin resistance, one gene which gathered our attention was *G6PC3* (fold change expression of MHNW = 1.00, MUNW = 0.31, MHOW = 1.10, MUOW = 0.79). Glucose 6 phosphatase enzymes have three subunits known as *G6PC*, *G6PC2*, and *G6PC3*, together known as G6PC family. Majorly, the GPC family works in association with fasting glucose levels [61,62]. *G6PC3* is ubiquitously expressed in cells and tissues, and hence, formerly known as “ubiquitously expressed G6Pase catalytic subunit related protein” (UGRP). Its mRNA has been reported to be more abundant than the other paralogous genes of *G6PC* [63,64]. *G6PC3* is well known for its expression in white blood cells, particularly neutrophils, and its deficiency leads to autosomal recessive disease SCN4 (severe congenital neutropenia type 4) [61,62,65]. This subunit of the paralogous gene of G6PC is lesser known for its regulation in blood glucose levels. To date, *G6PC3* known for regulation in neutrophils and deficiency will cause severe congenital neutropenia type 4 (SCN4). However, in our study, in the MUNW group, *G6PC3* has been downregulated, while upregulated in MHOW group suggesting its implication in nutrition-related pathways of insulin resistance.

Summarizing the present study, MHOW is a better metabolic health status with decreased WBC, RBC, thrombocytes, and inflammatory markers than MUNW. The increased blood levels of IL-6, TNF-α, and ferritin proved that inflammation is present in the unhealthy groups. Although ferritin is known as an important biomarker in iron-binding and transferring, it is also rising as an inflammatory marker in obesity [66]. The tendency and levels of inflammatory markers in all the groups suggest that metabolically healthy individuals have lower inflammation in comparison to metabolically unhealthy individuals. The lower inflammation is also proved at a genetic level where the ‘signaling of interleukins’ pathway was overexpressed in MUNW, while ‘metabolic pathways’ were overexpressed in MHOW. The significant difference in inflammation was observed only in IL-6 concentrations was due to the higher concentration of adiponectin, which acts as an anti-inflammatory protein, hence, limiting the expression and release of pro-inflammatory markers. This, therefore, helps in the development of a better metabolic profile [67]. Furthermore, the GO-biological pathways and GO terms showed expression of common processes suggesting MUNW and MHOW as the transition state between metabolically healthy and metabolically unhealthy health status.

## 5. Conclusions

To conclude our study, MHOW is indeed a better metabolic health status as predicted by GO biological processes and transcription factors; however, it is not a stable state. The major outcome from the study was a unique gene ‘*G6PC3*’ showing upregulation in MHOW and still unknown for its existence in insulin resistance. Moreover, the biochemical and genetic exploration has suggested that obesity (as a biological adaptation and MHOW and MUNW) are the transition states between metabolically healthy and metabolically unhealthy profiles—though, further studies are essential in this direction.

## Figures and Tables

**Table 1:** Anthropometric characteristics, energy intake, and energy expenditure of the study male participants.

S.No.	Characteristics	MHNW (*n* = 36)	MUNW (*n* = 36)	MHOW (*n* = 14)	MUOW (*n* = 25)
1	Age (y)	22.9 ± 4.1	23.7 ± 5.3	25.7 ± 7.5	25.9 ± 4.7
2	Height (cm)	171.7 ± 5.9	171.1 ± 6.8	170.7 ± 7.2	173.5 ± 5.4
3	Weight (Kg)	63.2 ± 6.3	65.5 ± 7.5	79.9 ± 11.0	83.8 ± 9.2
4	BMI (Kg/m^2^)	21.5 ± 1.7	22.3 ± 1.6	27.3 ± 1.9 *,#	27.8 ± 2.3 *,#
5	Fat Mass(Kg)	11.0 ± 2.8	12.4 ± 3.9	20.4 ± 4.6 *,#	22.6 ± 4.3 *,#
6	Fat Mass (%)	17.3 ± 3.8	18.6 ± 4.4	25.3 ± 2.6 *,#	26.8 ± 2.5 *,#
7	Fat Free Mass (Kg)	52.2 ± 4.9	53.1 ± 4.5	59.5 ± 6.7 *,#	61.2 ± 5.2 *,#
8	Muscle Mass (Kg)	49.5 ± 4.6	50.3 ± 4.3	56.5 ± 6.3 *,#	58.0 ± 4.9 *,#
9	Total Body Water (Kg)	34.8 ± 3.6	35.5 ± 3.0	41.5 ± 5.0 *,#	41.9 ± 4.2 *,#
10	BMR (kcal/day)	1561	1595	1804	1860
11	Energy Intake (kcal/day)	3112 ± 1133	3601 ± 1336	3358 ± 1574	3514 ± 1365
12	TEE (kcal/day)	3066 ± 849	3232 ± 980	3975 ± 1141	3789 ± 691
13	PAL	1.96	2.03	2.20	2.03

Values are expressed as mean ± SD. * *p* < 0.001 in comparison with MHNW, # *p* < 0.001 in comparison with MUNW, BMI, body mass index; BMR, basal metabolic rate; TEE, total energy expenditure; PAL, physical activity level; MHNW, metabolically healthy normal-weight; MUNW, metabolically unhealthy normal-weight; MHOW, metabolically healthy overweight/obese; MUOW, Metabolically unhealthy overweight/obese.

**Table 2:** Hematological profile of the study participants.

S.No.	Variables	MHNW	MUNW	MHOW	MUOW
1	Hb (g/dL)	14.7 ± 1.4	14.4 ± 1.5	14.5 ± 1.5	15 ± 1.4
2	WBC (10^9^/L)	7.4 ± 2.5	8.2 ± 2.4	7.5 ± 1.6	8.3 ± 2.5
3	Lymphocytes (10^6^/L)	36.9 ± 5.5	35.6 ± 8.5	34.0 ± 6.4	36.7 ± 6.6
4	RBC (M/mm^3^)	5.2 ± 0.5	5.0 ± 0.5	5.0 ± 0.5	5.5 ± 0.7 *
5	MCV (fL)	89.5 ± 6.8	89.7 ± 7.2	90.9 ± 4.3	87.7 ± 8.6
6	HCT (%)	46.5 ± 4.2	45.2 ± 4.6	45.5 ± 4.4	47.7 ± 5.0
7	MCH (pg/cell)	28.3 ± 2.5	28.4 ± 2.5	28.8 ± 1.4	27.6 ± 3.2
8	MCHC (g/dL)	29.9 ± 10.4	31.7 ± 0.9	31.8 ± 1.1	31.5 ± 1.3
9	THR (10^9^/L)	313 ± 80.5	352.3 ± 100	317.4 ± 48.8	326.3 ± 97.3

Values are expressed as mean ± SD.* *p* < 0.01 in comparison with MUNW and MHOW. Hb, hemoglobin; WBC, white blood cells; RBC, red blood cells; MCV, mean corpuscular volume; HCT, hematocrit; MCH, mean corpuscular hemoglobin; MCHC, mean corpuscular hemoglobin concentration; THR, thrombocytes.

**Table 3:** Top 5 upregulated and downregulated differentially expressed genes (DEGs) in the MUNW group in comparison to the MHNW group.

S. No.	Gene ID	Genes	Gene Name	*p*-Value	Differential Score
**Upregulated**					
1	720	*C4A*	Complement C4A (Rodgers Blood Group)	0.049	3.44
2	402	*EPCAM*	Epithelial Cell Adhesion Molecule	0.049	3.44
3	83,723	*FAM57B*	Family With Sequence Similarity 57 Member B	0.049	3.44
4	245,939	*DEFB128*	Defensin β 128	0.044	3.44
5	133,396	*IL31RA*	Interleukin 31 Receptor A	0.044	3.44
**Downregulated**					
1	55,278	*QRSL1*	Glutaminyl-TRNA Synthase (Glutamine-Hydrolyzing)-Like 1	0.048	−3.44
2	653,519	*GPR89A*	G Protein-Coupled Receptor 89A	0.047	−3.44
3	65,251	*ZNF649*	Zinc Finger Protein 649	0.047	−3.44
4	51,547	*SIRT7*	Sirtuin 7	0.044	−3.44
5	1017	*CDK2*	Cyclin Dependent Kinase 2	0.043	−3.44

**Table 4:** Top 5 upregulated and downregulated DEGs in the MHOW group in comparison to the MHNW group.

S. No.	Gene ID	Genes	Gene Name	*p*-Value	Differential Score
**Upregulated**					
1	80,778	*ZNF34*	Zinc Finger Protein 34	0.003	5.50
2	23,630	*KCNE1L*	Potassium Voltage-Gated Channel Subfamily E Regulatory Subunit 5	0.008	5.50
3	3280	*HES1*	Hes Family BHLH Transcription Factor 1	0.022	5.50
4	441,326	*FAM90A18*	Family With Sequence Similarity 90 Member A18, Pseudogene	0.044	5.50
5	1154	*CISH*	Cytokine Inducible SH2 Containing Protein	0	5.09
**Downregulated**					
1	38	*ACAT1*	Acetyl-CoA Acetyltransferase 1	0	−5.09
2	3109	*HLA-DMB*	Major Histocompatibility Complex, Class II, DM β	0	−5.09
3	26,995	*TRUB2*	TruB Pseuouridine Synthase Family Member 2	0.005	−5.09
4	79,912	*PYROXD1*	Pyridine Nucleotide-Disulphide Oxidoreductase Domain 1	0.023	−5.09
5	29,103	*DNAJC15*	DnaJ Heat Shock Protein Family (Hsp40) Member C15	0	−5.07

**Table 5:** Top 10 Enrichment pathway-GO Biological pathway for the MUNW group in comparison to the MHNW group.

S. No.	Term	Pathway/Term ID	Overlap	GSEA Library	Adjusted *p*-Value
1	JAK/STAT signaling pathway_Homo sapiens_P00038	P00038	12/14	Panther	0.031
2	Translation_Homo sapiens_R-HSA-72766	R-HSA-72766	85/151	Reactome	0.002
3	SRP-dependent cotranslational protein targeting to membrane_Homo sapiens_R-HSA-1799339	R-HSA-1799339	62/107	Reactome	0.009
4	Influenza Life Cycle_Homo sapiens_R-HSA-168255	R-HSA-168255	72/136	Reactome	0.043
5	Influenza Infection_Homo sapiens_R-HSA-168254	R-HSA-168254	77/147	Reactome	0.043
6	Major pathway of rRNA processing in the nucleolus_Homo sapiens_R-HSA-6791226	R-HSA-6791226	86/166	Reactome	0.043
7	rRNA processing_Homo sapiens_R-HSA-72312	R-HSA-72312	91/180	Reactome	0.048
8	Activation of the mRNA upon binding of the cap-binding complex and eIFs, and subsequent binding to 43S_Homo sapiens_R-HSA-72662	R-HSA-72662	35/58	Reactome	0.051
9	Cap-dependent Translation Initiation_Homo sapiens_R-HSA-72737	R-HSA-72737	61/114	Reactome	0.051
10	Eukaryotic Translation Initiation_Homo sapiens_R-HSA-72613	R-HSA-72613	61/114	Reactome	0.051

**Table 6:** Enriched Gene Ontology term in the MUNW group in comparison to MHNW.

S. No.	Enrichment Term	Pathway/Term ID	Overlap	GSEA Library	Adjusted *p*-Value
1	Cotranslational protein targeting to membrane	GO:0006613	65/110	GO	0.007
2	Protein targeting to ER	GO:0045047	65/111	GO	0.007
3	Establishment oof protein localization to endoplasmic reticulum	GO:0072599	67/115	GO	0.007
4	Srp-dependent cotranslational protein targeting to membrane	GO:0006614	63/108	GO	0.009
5	Protein localization to endoplasmic reticulum	GO:0070972	67/118	GO	0.014
6	Translation	GO:0006412	126/264	GO	0.252
7	Translational initiation	GO:0006413	72/139	GO	0.252
8	Protein targeting	GO:0006605	129/273	GO	0.304
9	Establishment of protein localization to organelle	GO:0072594	135/292	GO	0.549
10	Translational termination	GO:0006415	48/89	GO	0.549

**Table 7:** Enrichment pathway-GO Biological pathway for the MHOW group in comparison to the MHNW group.

S. No.	Term	Pathway/Term ID	Overlap	GSEA Library	Adjusted *p*-Value
1	The information-processing pathway at the IFN-β enhancer_Homo sapiens_h_pcafpathway	h_pcafpathway	18/29	Biocarta	0.058
2	Ribosome_Homo sapiens_hsa03010	hsa03010	62/137	KEGG	0.016
3	Influenza Life Cycle_Homo sapiens_R-HSA-168255	R-HSA-168255	68/136	Reactome	0.0003
4	SRP-dependent cotranslational protein targeting to membrane_Homo sapiens_R-HSA-1799339	R-HSA-1799339	56/107	Reactome	0.0003
5	Influenza Infection_Homo sapiens_R-HSA-168254	R-HSA-168254	71/147	Reactome	0.0004
6	Infectious disease_Homo sapiens_R-HSA-5663205	R-HSA-5663205	142/348	Reactome	0.0009
7	Major pathway of rRNA processing in the nucleolus_Homo sapiens_R-HSA-6791226	R-HSA-6791226	75/166	Reactome	0.0025
8	tRNA Aminoacylation_Homo sapiens_R-HSA-379724	R-HSA-379724	26/42	Reactome	0.0025
9	Nonsense-Mediated Decay (NMD)_Homo sapiens_R-HSA-927802	R-HSA-927802	52/106	Reactome	0.0025
10	Nonsense Mediated Decay (NMD) enhanced by the Exon Junction Complex (EJC)_Homo sapiens_R-HSA-975957	R-HSA-975957	52/106	Reactome	0.0025

**Table 8:** Enriched Gene Ontology term in the MHOW group in comparison to MHNW.

S. No.	Enrichment Term	Pathway/Term ID	Overlap	GSEA Library	Adjusted *p*-Value
1	Gene expression	GO:0010467	255/672	GO	0.001
2	Viral life cycle	GO:0019058	60/118	GO	0.001
3	Protein targeting to ER	GO:0045047	57/111	GO	0.001
4	Protein localization to endoplasmic reticulum	GO:0070972	59/118	GO	0.002
5	Establishment of protein localization to endoplasmic reticulum	GO:0072599	58/115	GO	0.002
6	SRP-dependent cotranslational protein targeting to membrane	GO:0006614	55/108	GO	0.002
7	Translation	GO:0006412	112/264	GO	0.002
8	Cotranslational protein targeting to membrane	GO:0006613	55/110	GO	0.002
9	Ncrna metabolic process	GO:0034660	135/332	GO	0.003
10	Nuclear-transcribed mrna catabolic process	GO:0000956	81/183	GO	0.005

## Data Availability

Data available in a publicly accessible repository.

## Supplementary Materials

The following are available online at https://www.mdpi.com/2073-4425/12/2/290/s1, Table S1: Prediction Equation for calculating basal metabolic rate (BMR) in Indian Males as per ICMR Expert Report [17], Table S2: Top significantly expressed Transcription Factors in MUNW in comparison to MHNW using Expression2Kinases tool, Table S3: Top significantly expressed Kinases in MUNW in comparison to MHNW using Expression2Kinases tool, Table S4: Top significantly expressed Transcription Factors in MHOW in comparison to MHNW using Expression2Kinases online tool, Table S5: Top significantly expressed Kinases in MHOW in comparison to MHNW using Expression2Kinases online tool, Table S6:Top Ten Hub Gene Analysis Performed Using Network Analyst tool in MUNW DEGs based on Degree in comparison to MHNW, Table S7:Top Ten Hub Gene Analysis Performed Using Network Analyst tool in MHOW DEGs based on Degree in comparison to MHNW, Table S8:Nutrition-Related Important Genes of MUNW using CytoscapeSoftware, Table S9:Nutrition-Related Important Genes of MHOW Using Cytoscape Software.
