# Supplementary material for: Deciphering Biochemical and Molecular Signatures Associated with Obesity in Context of Metabolic Health
Source: Genes (Basel). 2021 Feb 19;12(2):290. doi: 10.3390/genes12020290 (PMC7923210; doi:10.3390/genes12020290)
Supplement: Supplementary file 1 [file genes-12-00290-s001.pdf]

Supplementary Material

Table 1. Prediction Equation for calculating basal metabolic rate (BMR) in Indian Males as per ICMR Expert Report (17)

Age (years)	Prediction equation	Correlation Coefficient	SD
18-30	14.5 x Body Weight (kg) + 645	0.65	151
30-60	10.9 x Body Weight (kg) + 833	0.60	164
>60	12.6 x Body Weight (kg) + 463	0.79	146

Table 2. Top significantly expressed Transcription Factors in MUNW in comparison to MHNW using Expression2Kinases tool

S.No.	Term	Gene name	Overlap	p-value
1	AHR	Aryl Hydrocarbon Receptor	25/42	0.003
2.	EED	Embryonic Ectoderm Development	47/91	0.004
3.	ATF2	Activating Transcription Factor 2	108/237	0.006
4.	BRF2	BRF2, RNA Polymerase III Transcription Initiation Factor Subunit	32/60	0.008
5.	YY1	YY1 Transcription Factor	58/120	0.009

Table 3. Top significantly expressed Kinases in MUNW in comparison to MHNW using Expression2Kinases tool

S.No.	Term	Gene name	Overlap	p-value
1	LRRK2	Leucine Rich Repeat Kinase 2	07/07	0.016
2.	TXK	TXK Tyrosine Kinase	03/03	0.052
3.	TSSK6	Testis Specific Serine Kinase 6	03/03	0.052

Table 4. Top significantly expressed Transcription Factors in MHOW in comparison to MHNW using Expression2Kinases online tool

.No.	Term	Gene name	Overlap	Adjusted p-value
1	ESR1	Estrogen Receptor 1	307/871	0.0099
2.	FOXP3	Forkhead Box P3	136/350	0.0099
3.	ESRRB	Estrogen Related Receptor Beta	41/84	0.0118
4.	GATA1	GATA Binding Protein 1	34/69	0.0260
5.	NFKB1	Nuclear Factor Kappa B Subunit 1	98/254	0.0422
6.	POU5F1	POU Class 5 Homeobox 1	126/338	0.0422
7	TP53	Tumor Protein P53	219/628	0.0505

Table 5. Top significantly expressed Kinases in MHOW in comparison to MHNW using Expression2Kinases online tool

S.No.	Term	Gene name	Overlap	p-value
1	LCK	Lymphocyte Cell-Specific Protein-Tyrosine Kinase	40/96	0.006
2.	HCK	Hemopoietic Cell Kinase	19/41	0.015
3.	MAP3K1	Mitogen-Activated Protein Kinase Kinase Kinase 1	8/13	0.015
4.	CHEK1	Checkpoint Kinase 1	52/137	0.017
5.	SIK2	Salt Inducible Kinase 2	03/03	0.025

Table 6. Top Ten Hub Gene Analysis Performed Using Network Analyst tool in MUNW DEGs based on Degree in comparison to MHNW

S. No.	Gene ID	Label	Gene Name	Degree	Bet	p-value	Fold change
UPREGULATED							
1	Q15717	ELAVL1	ELAV Like RNA Binding Protein 1	753	1634759	0.039	-3.37
2	P61956	SUMO2	Small Ubiquitin-Like Modifier 2	496	650443.4	0	-3.42
3	Q9NRC8	SIRT7	Sirtuin 7	287	210199.5	0.044	-3.44
4	Q13616	CUL1	Cullin 1	258	135914.8	0	-3.05
5	P24941	CDK2	Cyclin Dependent Kinase 2	255	168311.2	0.044	-3.44
DOWNREGULATED							
1	P46976	GYG1	Glycogenin 1	1	0	0	-2.99
2	Q8NEM7	FAM48A	SPT20 Homolog, SAGA Complex Component	1	0	0.047	-2.33
3	P84101	SERF2	Small EDRK-Rich Factor 2	1	0	0	-2.94
4	Q9UK76	HN1	Hematological And Neurological Expressed 1 Protein	1	0	0	-3.44
5	Q99653	CHP1	Calcineurin Like EF-Hand Protein 1	1	0	-	

Table 7. Top Ten Hub Gene Analysis Performed Using Network Analyst tool in MHOW DEGs based on Degree in comparison to MHNW

	Gene ID	Label	Gene Name	Degree	Bet	p-value	Fold change
	UPREGULATED						
1	P61956	SUMO2	Small Ubiquitin-Like Modifier 2	425	616100.7	0	-2.33
2	Q92905	COPS5	COP9 Signalosome Subunit 5	261	199585.3	0	-3.57
3	Q86VP6	CAND1	Cullin Associated And Neddylation Dissociated 1	236	156668	0	-2.90
4	Q13616	CUL1	Cullin 1	210	122587.7	0	3.070
5	P55072	VCP	Valosin Containing Protein	161	182152.4	0	3.239
DOWNREGULATED							
1	Q8N584	TTC39C	Tetratricopeptide Repeat Domain 39C	1	0	0	-2.94
2	Q9NUH8	TMEM14B	Transmembrane Protein 14B	1	0	0	-3.44
3	O95822	MLYCD	Malonyl-CoA Decarboxylase	1	0	0	-2.34
4	P19113	HDC	Histidine Decarboxylase	1	0	0.007	-3.41
5	Q8IZF4	GPR114	G Protein-Coupled Receptor 114	1	0	0.028	-2.48

Table 8. Nutrition Related Important Genes of MUNW using Cytoscape Software

S. No.	Gene ID	Genes	Gene Name	Closeness Centrality	Degree	Betweeness Centrality
1	5296	PIK3R2	Phosphoinositide-3-Kinase Regulatory Subunit 2	0.347201	20	0.088625
2	5781	PTPN11	Protein Tyrosine Phosphatase, Non-Receptor Type 11	0.289756	14	0.03689
3	3717	JAK2	Janus Kinase 2	0.280149	13	0.022468
4	7124	TNF	Tumor Necrosis Factor	0.292261	10	0.048044
5	92579	G6PC3	Glucose-6-Phosphatase Catalytic Subunit 3	0.276031	9	0.022568

Table 9. Nutrition Related Important Genes of MHOW Using Cytoscape Software

S. No	Gene ID	Genes	Gene Name	Closeness Centrality	Degree	Betweeness Centrality
1	5291	PIK3CB	Phosphatidylinositol-4,5-Bisphosphate 3-Kinase Catalytic Subunit Beta	0.332577	16	0.039
2	207	AKT1	AKT Serine/Threonine Kinase 1	0.295326	13	0.009
3	5330	PLCB2	Phospholipase C Beta 2	0.334703	11	0.039
4	5595	MAPK3	Mitogen-Activated Protein Kinase 3	0.289266	11	0.007
5	7124	TNF	Tumor Necrosis Factor	0.281274	10	0.007
6	32	ACACB	Acetyl-CoA Carboxylase Beta	0.3794	9	0.030
